# Comparative Assessment of Substrates and Activity Based Probes as Tools for Non-Invasive Optical Imaging of Cysteine Protease Activity

**DOI:** 10.1371/journal.pone.0006374

**Published:** 2009-07-28

**Authors:** Galia Blum, Robby M. Weimer, Laura E. Edgington, Walter Adams, Matthew Bogyo

**Affiliations:** 1 Department of Pathology, Stanford University School of Medicine, Stanford, California, United States of America; 2 Department of Biomedical Imaging, Genentech Inc., South San Francisco, California, United States of America; 3 Department of Microbiology and Immunology, Stanford University School of Medicine, Stanford, California, United States of America; Johns Hopkins School of Medicine, United States of America

## Abstract

Recent advances in the field of non-invasive optical imaging have included the development of contrast agents that report on the activity of enzymatic targets associated with disease pathology. In particular, proteases have proven to be ideal targets for development of optical sensors for cancer. Recently developed contrast agents for protease activity include both small peptides and large polymer-based quenched fluorescent substrates as well as fluorescently labeled activity based probes (ABPs). While substrates produce a fluorescent signal as a result of processing by a protease, ABPs are retained at the site of proteolysis due to formation of a permanent covalent bond with the active site catalytic residue. Both methods have potential advantages and disadvantages yet a careful comparison of substrates and ABPs has not been performed. Here we present the results of a direct comparison of commercially available protease substrates with several recently described fluorescent ABPs in a mouse model of cancer. The results demonstrate that fluorescent ABPs show more rapid and selective uptake into tumors as well as overall brighter signals compared to substrate probes. These data suggest that the lack of signal amplification for an ABP is offset by the increased kinetics of tissue uptake and prolonged retention of the probes once bound to a protease target. Furthermore, fluorescent ABPs can be used as imaging reagents with similar or better results as the commercially available protease substrates.

## Introduction

**Figure 1:**
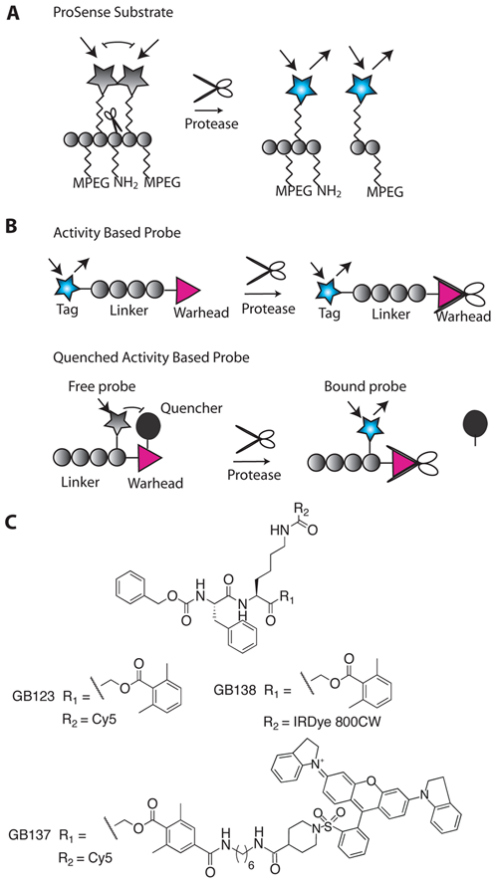
Fluorescent protease probes for non-invasive imaging. (A) Schematic diagram illustrating activation of the ProSense imaging probes by a target protease. The main backbone is made up of PEGylated poly-lysine modified with fluorophores that are quenched by close proximity (gray stars). Upon cleavage by a protease at free lysine residues, smaller fragments containing the unquenched fluorophore (blue stars) are released. (B) Schematic of fluorescent activity based probes labeling a target protease. In both examples, the probe covalently binds in the active site of the protease target forming a permanent bond between the probe and the protease. In the top scheme, the probe contains a fluorescent reporter (blue star) that emits fluorescence even in the absence of protease. In the bottom scheme, the fluorophore (gray star) on the ABP is quenched by proximity to a quenching group (black circle) that is lost upon covalent modification of the target protease. (C) Structures of the ABPs used in this study. The probes GB123 and GB138 do not contain a quenching group and are therefore always fluorescent. GB137 contains a Cy5 fluorophore that is quenched by proximity to the QSY quenching group.

The past decade has seen a dramatic increase in the number of new technologies that are available for applications in molecular imaging and disease monitoring. The field of optical fluorescence imaging has begun to show promise as a method that may soon have significant clinical value. At the heart of all new imaging methods is the need for contrast agents that provide a more precise picture of distinct molecular events as they happen *in vivo*. Some of the most recently developed classes of contrast agents are so-called “smart probes” that produce signal in response to a specific enzyme mediated reaction. Because increased protease activity has been shown to be associated with the pathogenesis of a number of human diseases including cancer, atherosclerosis and neurodegenerative diseases, significant efforts have been made to develop molecular sensors of protease activity. A large number of reagents have been built around reporter substrates that, when cleaved by a protease, produce a fluorescent signal (for review see[1], [2]). These reagents include large, polymer-based quenched fluorescent substrates that are cleaved in multiple locations to produce fluorescent products (**Fig. 1A**). As an alternative to substrates, fluorescent activity based probes have also been reported 3, 4. Unlike substrates, these reagents label target proteases through the formation of a covalent bond with the active site cysteine (**Fig. 1B**). Because substrates and ABPs have highly distinct mechanisms of action, a direct comparison of these two classes of agents in a relevant biological system would be valuable for understanding the strengths and weaknesses of each method.

Protease activity has historically been biochemically dissected using protein and peptide substrates. One of the most common tools used to monitor protease activity is a fluorogenic peptide substrate. Various classes of fluorescent substrates have been described, including those with reporters that change fluorescent properties upon peptide bond hydrolysis, substrates containing quencher or FRET reporter pairs that become separated by protease cleavage of the peptide backbone and substrates that are retained within cells when cleaved by a protease [5], [6], [7], [8]. These substrate types of have been designed using short peptide sequences [5], [9] or using fluorophores or fluorescent peptide sequences tethered to a large polymer or dendramer backbone [7], [8]. Polymer-based substrates are internally quenched by the high density of fluorophores loaded onto the backbone structure and are designed to produce fluorescent products upon protease processing. One such class of polymer-based reagents, ProSense® (VisEn medical), is commercially available and an analog of this probe family is currently moving towards early stage human clinical trails (VisEn Medical Press Release June 16, 2008).

For all classes of substrates, selectivity for a given protease target is controlled by the peptide recognition sequence. While some proteases show a high degree of selectivity for a given sequence (i.e. caspases which require a P1 aspartic acid) and can be targeted with relatively selective substrates, others show high promiscuity and are able to cleave a wide variety of peptide sequences. Unfortunately, with more than 500 proteases in the human genome [10], it remains extremely difficult to control selectivity of substrate cleavage *in vivo*. In addition, the overall pharmacodynamic properties of the substrate ultimately dictate which proteases come into contact with the reporter and to what extent a substrate will accumulate in a given tissue or organ. Regardless of the drawbacks of selectivity, one of the major benefits of using a substrate as a reporter is that a single active protease can process many substrates, thus leading to signal amplification over time. This amplification has been proposed to be a highly beneficial property of substrate based imaging agents yet, the real contribution of signal amplification has not been evaluated.

As an alternative to substrate based imaging agents, activity based probes have recently been shown to have value for non-invasive optical imaging applications [4]. One of the major advantages of ABPs is that they covalently bind to a target protease, allowing direct biochemical analysis of targets after *in vivo* imaging has been performed. Furthermore, the selectivity of an ABP can be controlled both by the peptide selectivity sequence and the type of reactive functional group or “warhead” used on the probe. Thus, it is possible to generate probes that have exceedingly high selectivity for a small number of related proteases, as has been demonstrated for the caspases and cysteine cathepsins [11], [12], [13], [14]. In addition, since ABPs tend to be small molecules with relatively short half-lives *in vivo* they have the potential to circulate quickly and be rapidly cleared resulting in the production of high contrast images. However, since ABPs are also inhibitors of their target enzymes, they bind only a single target protease molecule and fluorescent signals are not amplified by multiple processing events. Therefore, a direct comparison of substrates and ABPs should provide information regarding the value of signal amplification for optical imaging contrast reagents.

For this reason, we decided to perform a direct comparison of a series of substrates and ABPs that have been independently validated as imaging agents for the cysteine cathepsins. Specifically, we compared the commercially available ProSense polymer-based substrates to the previously described fluorescently quenched and non-quenched probes GB123, GB138 and GB137 in a mouse model of cancer. For this study we co-injected substrates and probes carrying non-overlapping fluorescent reporters into the same animal and then compared fluorescent images over time. In addition we evaluated probe uptake as well as overall tissue distribution *in vivo*. To rule out the possibility of inhibition of substrate signal by co-injection of the probes, we also performed the comparison in separate animals with similar outcome. Overall, our results indicate that the large polymer-based substrates show slow uptake into tumors, have relatively high background in organs such as liver and spleen and produce overall weaker signals compared to the fluorescent ABPs. Based on these findings we conclude that slow uptake and rapid diffusion of the fluorescent products of substrates prevent signal amplification from dramatically enhancing imaging signals and therefore ABPs can be used with similar or better results.

## Results

In order to directly compare substrate and ABP imaging agents, we evaluated both classes of reagents in a simple xenograft mouse model of cancer. For ABPs, we chose to use several recently reported probes that target the cysteine cathepsins [3], [4] (**Fig. 1C**). These include the fluorescently quenched probe GB137 and the non-quenched probes GB123 and GB138. All of these reagents have the same general scaffold and reactive acyloxymethyl ketone (AOMK) warhead group. The primary difference is the presence of a QSY21 quenching group on the acyloxy leaving group of GB137 and the use of an IR800 fluorophore in place of Cy5 on GB138. As an initial comparison we chose to evaluate the commercially available ProSense® (VisEn Medical) substrates because they also have been shown to be activated by cathepsins. These reagents consist of a pegylated poly-lysine backbone derivatized with fluorophores. The high density of fluorophores results in a polymer that is non-fluorescent due to self-quenching. Upon cleavage of the poly-lysine backbone by a protease, fluorescent fragments are released (**Fig. 1A**). Although these reagents are effectively processed by multiple cathepsins, recent data suggest that they can also be processed by other classes of proteases (VisEn Medical website). Since substrates and ABPs use fluorophores that emit light in the near infrared region, it is possible to monitor their activation using whole body, non-invasive imaging methods.

**Figure 2:**
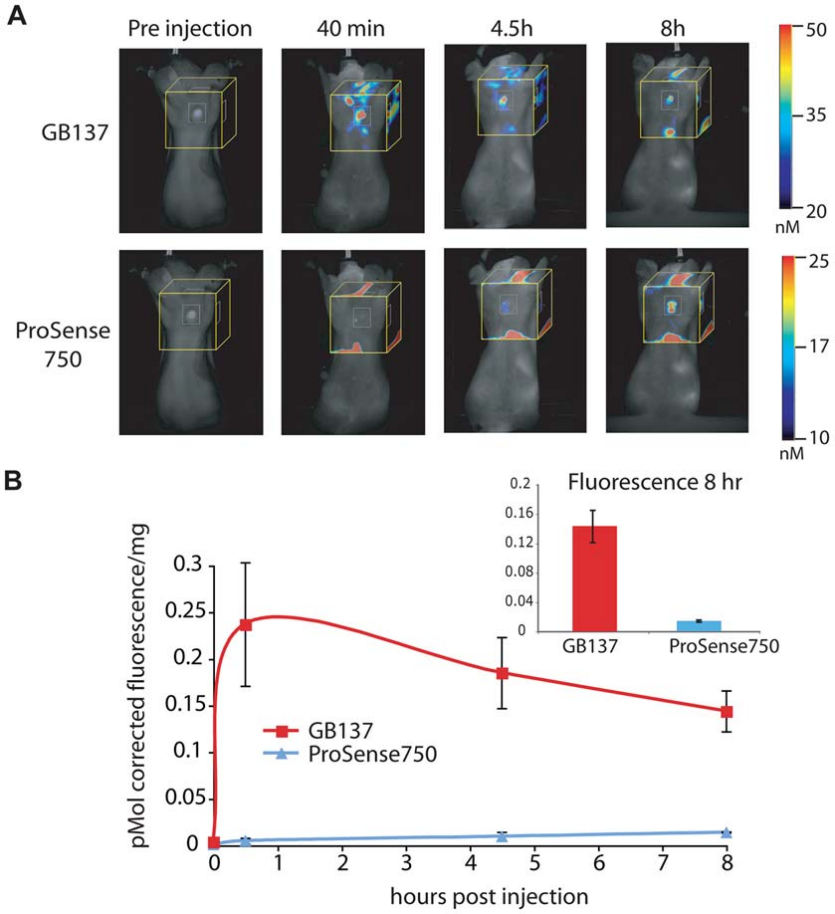
Comparison of GB137 and ProSense750 using non-invasive optical imaging methods. (A) Images of GB137 and ProSense 750 fluorescence in mice bearing MDA-MB 231 MFP tumors. Fluorescent images of live mice were taken at various time points after injection using an FMT1 imaging system. Representative pictures of the same mouse scanned over time in the 680 nm channel (GB137) and 750 nm channel (Prosense750) are shown. Fluorescent tomography scans (yellow box) are overlaid on an epifluorescent image. The colorometric scale bars indicate the amount of fluorescence in the tomography box. (B) Quantification of total fluorescence in tumors. Fluorescence in tumors was measured in the indicated area (white box) and normalized to tumor weight. Mean fluorescence and standard error normalized to tumor weight for each channel is plotted relative to time after probe injection. Insert shows tumor fluorescence (in pmol) normalized to tumor weight at the 8 hour time point for GB137 and ProSense750 treated mice. GB137 fluorescence was corrected to account for the non-optimal filter set of the FMT1 (see methods and Fig. S1).

We initially evaluated the ProSense750 probe and GB137 in nude mice bearing human MDA-MB 231 MFP tumors (**Fig. 2**). Since GB137 emits 666 nM light while ProSense750 emits 750 nM light, we could inject both probes into the same animal and monitor each probe individually using different filter sets. Probes were co-injected via tail vein and overall fluorescence was monitored using fluorescence tomography (FMT1, VisEn Medical) at various time points (**Fig. 2A**). By using the FMT imaging system we were able to visualize probe distribution in tumors as well as surrounding normal tissues throughout the entire depth of the animal and obtain quantitative readouts of total fluorescence in a given region of interest. Images obtained at early time points after probe injection (i.e. 40 min) show rapid and specific activation of the GB137 probe with no signals observed for ProSense750. The ProSense750 probe only showed measurable activation at the 8 hr time point, in agreement with protocols for these agents in which imaging is performed 24 hrs after probe injection[8]. In addition, we quantified tumor fluorescence and corrected the signal intensity based on the fluorophore used. Since the Cy5 and IR800 dyes used on the ABPs do not fluoresce at a wavelength that is optimally measured using the standard FMT filter set, we measured the total fluorescence intensity of a probe standard to determine a scale factor that could be used to accurately quantify the fluorescent signal for the ABPs (**Fig. S1**). The resulting analysis confirmed that GB137 fluorescence was brighter than the ProSense750 signal at the 8 hour time point (**Fig. 2B**).

**Figure 3:**
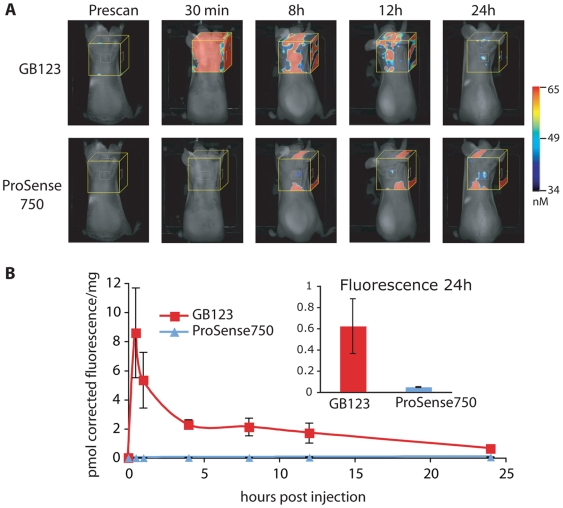
Comparison of GB123 and ProSense750 using non-invasive optical imaging methods. (A) Images of GB123 and ProSense 750 fluorescence in mice bearing MDA-MB 231 MFP tumors. Fluorescent images of live mice were taken at various time points after injection using an FMT1 imaging system. Representative pictures of the same mouse scanned over time in the 680 nm channel (GB123) and 750 nm channel (Prosense750) are shown. Fluorescent tomography scans (yellow box) are overlaid on an epifluoerscent image. The colorimetric scale bars indicate the amount of fluorescence in the tomography box. (B) Quantification of total fluorescence in tumors. Fluorescence in tumors was measured in the indicated area (white box) and normalized to tumor weight. Mean fluorescence normalized to tumor weight and standard error for each channel were plotted relative to time after probe injection. Insert shows the tumor fluorescence (in pmol) normalized to weight at the 24 hour time point of GB123 and ProSense750 treated mice. GB123 fluorescence was corrected to account for the non-optimal filter set of the FMT1 (see methods and Fig. S1).

We next compared the non-quenched ABP GB123 to the ProSense750 substrate in the same xenograft tumor model (**Fig. 3A**). Since GB123 is not quenched, it showed the expected high background signals at the early time points as previously reported [4]. However, we observed specific accumulation of the probe in tumors at the 12 hour and 24 hour time points. ProSense750 again showed specific tumor labeling after 8 hours and had the brightest signal 24 hours after probe injection. Quantification of total tumor fluorescence at the 24 hour time point indicated an overall 10–12 fold brighter probe signal for the ABP compared to the substrate (**Fig. 3B**).

**Figure 4:**
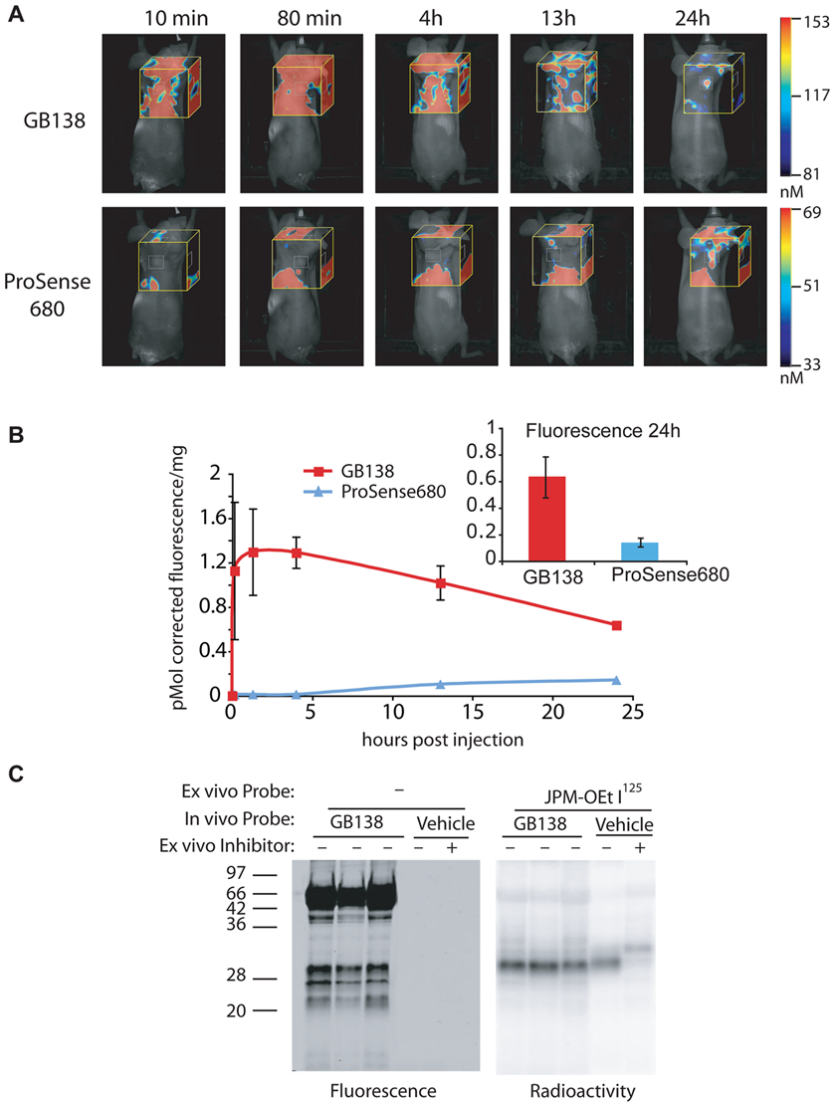
Comparison of GB138 and ProSense680 using non-invasive optical imaging methods. (A) Images of GB138 and ProSense 680 fluorescence in mice bearing MDA-MB 231 MFP tumors. Fluorescent images of live mice were taken at various time points after injection using an FMT1 imaging system. Representative pictures of the same mouse scanned over time in the 750 nm channel (GB138) and 680 nm channel (Prosense680) are shown. Fluorescent tomography scans (yellow box) are overlaid on an epifluoerscent image. The colorimetric scale bars indicate the amount of fluorescence in the tomography box. (B) Quantification of total fluorescence in tumors. Fluorescence in tumors was measured in the indicated area (white box) and normalized to tumor weight. Mean fluorescence and standard error normalized to tumor weight for each channels were plotted relative to time after probe injection. Insert shows tumor fluorescence (in pmol) normalized to weight at the 24 hour time point for GB138 and ProSense680 treated mice. GB138 fluorescence was corrected to account for the filter set of the FMT1 which is not optimal for the IR800 fluorophore (see methods and Fig. S1). (C) Residual activity of cathepsins in tumors from GB138/ProSense680 treated mice. Residual cathepsin activity was either directly labeled by addition of I^125^ JPM-OEt for 45 minutes, or samples were pretreated for 15 minutes with GB111-NH_2_ (a broad spectrum cathepsin inhibitor [3]) prior to I^125^ JPM-OEt labeling. Samples were analyzed by SDS-PAGE, followed by fluorescent scanning of the gel using an Odyssey scanner (left panel) followed by autoradiography (right panel).

To determine if the fluorescent tag had a direct effect on probe uptake and overall signal strength we co-injected tumor-bearing mice with the IR800 labeled ABP GB138 and the ProSense680 probe (**Fig. 4A**). Since the GB138 probe is not quenched, it showed high background labeling due to free probe circulation, however tumor specific labeling was observed by 12 hours after probe injection. Like GB123, GB138 showed good signal accumulation in the tumor with high tumor to background ratios at the late time points. In addition, the GB138 probe produced a signal in tumors that was more than 3 times brighter than the ProSense680 substrate at the 24 hour time point (**Fig. 4B**). Since the ABPs also act as covalent inhibitors, we wanted to make sure that the ProSense signals were not reduced as the result of target inhibition by the ABP. Therefore, we monitored the levels of active cathepsin B in tumors from probe and control treated animals. Analysis of tumor tissues from mice treated with GB138 at the same dose used for imaging studies confirmed that the fluorescent probe efficiently labeled active cathepsin B and L. However, tissues from both probe and control treated animals showed similar levels of residual active cathepsin B, as measured by *ex vivo* labeling of tissue extracts using a radiolabeled probe (**Fig. 4C**). These data suggest that, at the doses used for imaging, GB138 only labels a sub-population of active cathepsins, leaving the majority of the active enzyme intact.

**Figure 5:**
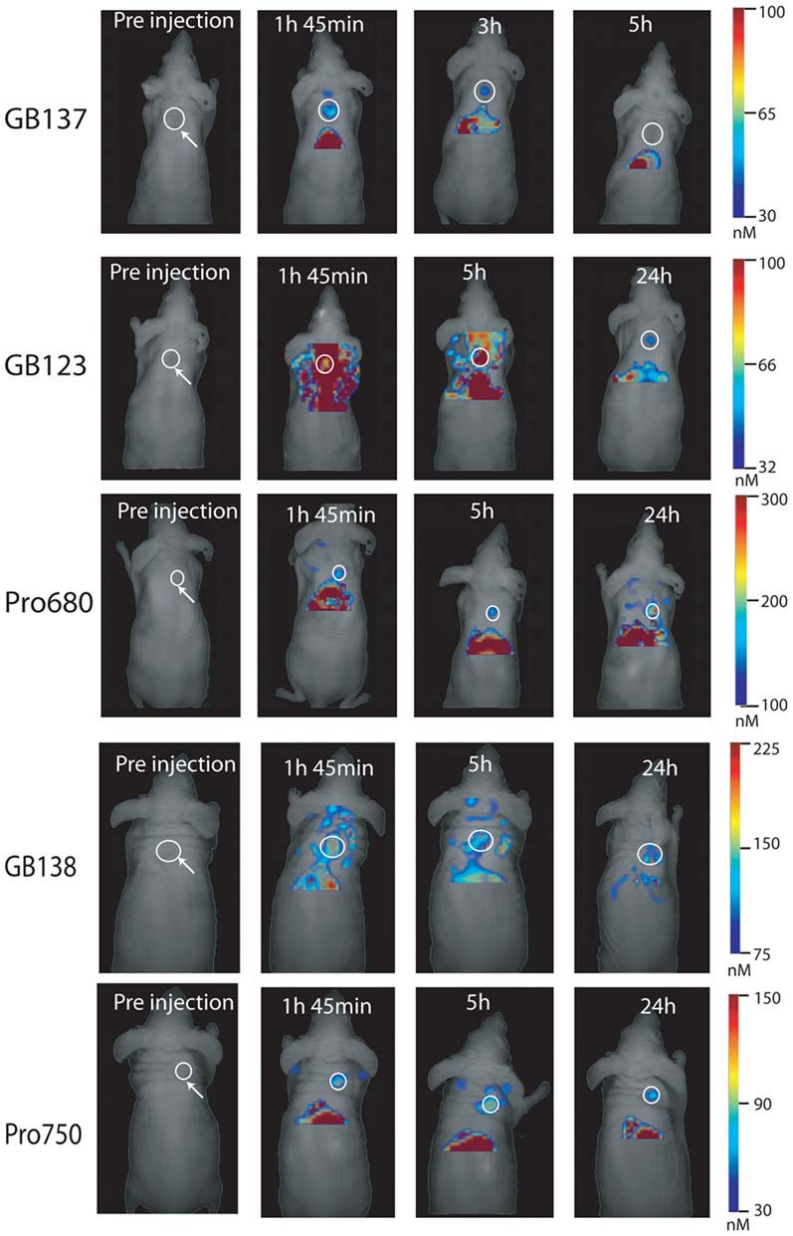
Comparison of all ABPs and substrate probes in parallel in separate mice. Fluorescence images of live mice bearing C2C12ras tumors treated with GB137, GB123, GB138, ProSense750 and ProSense680. Images were taken at various time points after injection using the FMT2500 imaging system. Representative pictures of each mouse scanned over time in the 680 nm or 750 nM channel are presented. Fluorescent tomography scans in color are overlaid over an epifluorescent image in black and white. The colorimetric scale bars indicate nm of fluorescence. White circles indicate the location and rough size of individual tumors. These circles do not indicate the area used for quantification of tumor fluorescence in figure 6.

**Figure 6:**
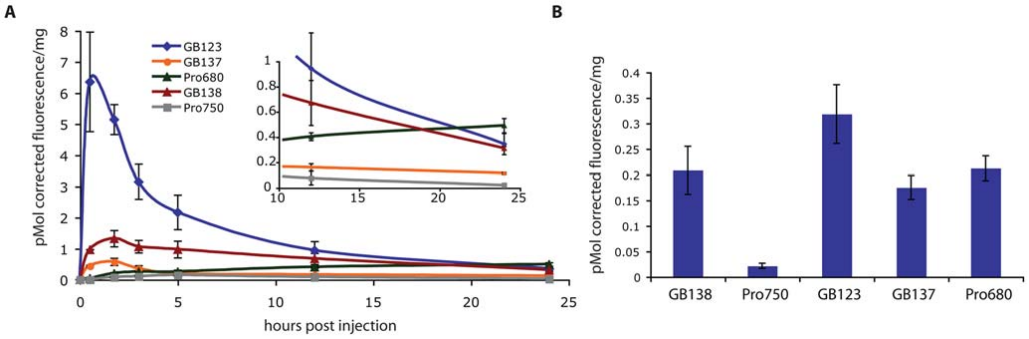
Quantification of tumor fluorescence in mice treated in parallel with ABPs and ProSense substrates. (A) Plot of fluorescence in tumors from mice shown in Figure 5 at various time points after probe injection. The fluorescence in tumors was measured using a constant ROI and normalized to tumor weight. Mean fluorescence and standard error is plotted relative to time after probe injection. (B) Total tumor fluorescence in tumors excised 24 hours after probe injection and imaged *ex vivo* using the FMT2500 system. Mean fluorescence with standard error is shown.

To further confirm that co-injection of the ABP probes did not artificially alter the ability of the ProSense probes to function, we performed imaging studies in which mice were separately injected with each of the probes. For these studies we compared all substrates and ABPs in mice carrying tumors derived from the Ras transformed mouse myoblast cell line C2C12 (**Fig. 5**). We have previously shown that these tumors grow faster and generally have higher cathepsin activity than the MDA-MB-231 cells [4]. In addition we performed imaging using the newly released FMT2500, which allows tomographic imaging of animals without the need for a density matching fluid. Direct comparison of probe labeling in this model confirmed our earlier results. Specifically, we found that only the quenched ABP GB137 provided specific probe signal in tumors at the early time points. Again, the non-quenched ABPs and the ProSense680 probe showed good contrast in tumors at the 5 and 24 hr time points. Quantification of the tumor fluorescence normalized to tumor weight indicated that the Cy5 labeled ABP GB123 provided the brightest signal while GB138 and GB137 produced similar signal accumulation to the ProSense680 probe (**Fig. 6A, B**). Interestingly, although the ProSense750 probe provided higher contrast in tumors at the early time points compared to ProSense680, it produced substantially weaker signal when compared to any of the other probes.

**Figure 7:**
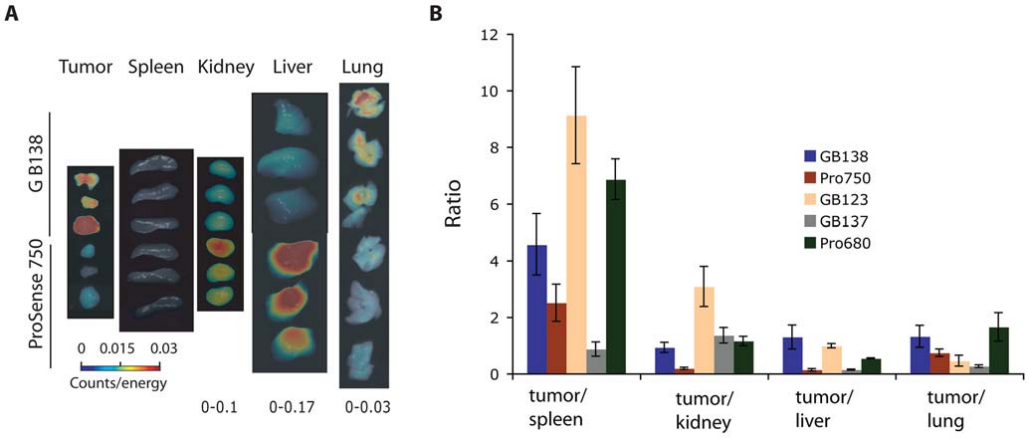
Evaluation of probe distribution and tissue specificity. (A). Representative images of tissues from ProSense750 and GB138 treated mice analyzed by *ex vivo* imaging using the FMT2500 imaging system. The relative scale for each set of tissues is indicated at the bottom. (B) Quantification of accumulation of probes in tumors relative to other major organs for each of the probes tested. The fluorescence measurements were corrected for Cy5 and IR800 since the filter sets on the FMT system are not optimized for their maximum excitation/emission wavelengths (see methods and Fig. S1).

Finally, we removed tissues from mice after the 24 hr time point and performed *ex vivo* imaging of the organs (**Fig. 7A**). This allowed us to confirm that the difference in tumor fluorescence observed in the live animals was consistent with the signals observed in the tumor tissues after removal. Furthermore, it allowed us to determine the relative uptake of the probes into various major organs to determine if there was a difference in background and probe clearance (**Fig. 7B**). These data indicate that in all tissues other than lung, the ABPs showed reduced uptake compared to the ProSense substrates. Specifically we observed high signal in the kidney and liver of ProSense treated mice.

## Discussion

Recent advances in the development of new classes of optical sensors have had a profound impact on the field of non-invasive fluorescence imaging. While there have been significant improvements in the instrumentation available for optical imaging applications, even the most sensitive instruments cannot provide molecular information that is critical for disease diagnosis and detection using optical methods. Perhaps one of the most promising new approaches for the development of optical contrast agents is the development of “smart probes” that produce a specific signal as the result of the action of a given enzymatic target. The cysteine cathepsins have proven to be involved in the regulation and mis-regulation of a number of key biological processes associated with a wide range of diseases, including cancer [15]. As a result, a number new methods have been developed that take advantage of increased protease activity in and around a diseased tissue of interest.

Several new classes of contrast agents have been designed to target proteases *in vivo*. The majority of these agents are designed to act as substrates for a protease that, when hydrolyzed at a given peptide bond, either relieve the quenching of a reporter, produce a fluorescent byproduct or become retained in tissues at the site of proteolysis [5], [6], [7], [8]. As an alternative to substrate-based reporters, we have recently described the use of small molecule activity based probes that carry a fluorescent reporter [3], [4]. These probes bind to the active form of a protease and become permanently bound to the active site nucleophile. ABPs can also be made in a quenched form so that the fluorescent signal is only produced upon reaction with the target protease [3]. The major difference between ABPs and substrates is that the ABP binds to a single target protease and inactivates it. Substrates, on the other hand, are processed and turned over by the protease, leaving the enzyme active, thus allowing for a potential amplification of signal as more substrate molecules are processed. Because there had not been a careful analysis of the benefits of signal amplification of substrates *in vivo*, we decided to carry out a study to directly compare commercially available fluorescent substrates to our fluorescently labeled activity based probes.

In this study we demonstrate in two different tumor types that the large, polymer-based ProSense substrates are in fact less bright than the small molecule ABPs. We believe that the reason why amplification is not leading to a significantly brighter signal is that these substrates are large molecules (>100,000 MW) that circulate slowly and diffuse slowly into tissues and cells. Once at the site of proteolysis, they are processed to release small fluorescent fragments that are then able to diffuse away from the site much more readily. As a result, the amplification of signal at the site of proteolysis does not have a dramatic enhancing effect for imaging. Furthermore, the resulting fluorescent fragments are most likely cleared through the liver and kidneys. This may explain why we see significantly higher accumulation of fluorescent signal in the kidney and liver of ProSense treated animals. Regardless of the interpretation of these results, the data clearly demonstrate that ABPs are able to produce signals that are at least as bright as the signals observed for substrate probes.

As an alternative to large-polymer-based probes, there has also recently been a report of small quenched peptide substrates that were designed based on our ABP scaffold as well as on selective inhibitor scaffolds in the patent literature [9]. These compounds should display increased uptake and clearance similar to that observed for our ABPs. We therefore anticipate that these reagents will provide rapid activation, however, they are likely to suffer from rapid clearance. In addition, all substrates suffer from the significant drawback that they generally lack selectivity. Since the only way to control selectivity is through the peptide recognition sequence, it is often difficult to generate substrates that show selective processing by a single protease or even family of proteases. ABPs have the advantage of using highly specific functional group chemistry that dramatically restricts the potential targets of the probes (i.e. only cysteine proteases will be covalently modified by an AOMK probe). In addition, ABPs remain bound to the target protease, leading to prolonged retention at the site of interest (i.e. the tumor) and allowing subsequent assessment of selectivity of target labeling using biochemical methods.

One of the potential drawbacks of using an ABP is the fact that target binding leads to inhibition of enzymatic activity. To address this issue, we have evaluated the extent of cysteine cathepsin inhibition upon treatment of animals at the dose of the probe used for imaging experiments. We show here that after treatment of animals with the ABP probe GB138, we are able to see strong residual labeling of target cathepsins by *ex vivo* addition of a radiolabeled ABP. Thus, we find that there is virtually no reduction in cysteine cathepsin protease activity compared to mice treated with vehicle control. This suggests that, at the doses used to generate optical imaging data, the probes are only modifying and inhibiting a small percentage of the total pool of active cathepsins. Therefore, we do not anticipate that probe labeling will in any way impact disease progression or lead to toxic effects as the result of inhibition of target proteases. Interestingly, we found the ProSense signals were somewhat reduced when we co-injected ABPs and substrates, suggesting that ABP inhibition of target proteases may have reduced the ProSense signals to some degree. This also suggests that the ProSense reagents are only measuring a small percentage of active proteases, as their signal can be reduced without inhibiting a significant portion of the total active cathepsins.

Taken together, our results suggest that both substrates and ABPs are viable tools for imaging protease activity using non-invasive imaging methods. However, our data suggest that the assumption that substrates produce brighter signal as the result of signal amplification is not valid, at least in the tumor models we have analyzed here. We also demonstrate that, because of their small size and overall rapid clearance *in vivo*, ABPs produce high contrast images more rapidly and with lower background retention in tissues such as liver and kidney compared to the ProSense substrates. These properties coupled with the fact that ABPs can be used to biochemically monitor protease targets after imaging make them preferential to substrates for some imaging applications. We are currently working to apply these and other classes of fluorescent ABPs in additional mouse models of human disease.

## Materials and Methods

### Fluorescent probes

The ProSense probes used in this study were purchased and used as directed by the manufacturer (VisEn Imaging, Inc.). The fluorescent ABPs were all synthesized and purified as described previously 3, 4.

### Non-invasive imaging of tumor bearing mice

All animal experiments were approved by the Stanford Administrative Panel on Animal Care and strictly followed their specific guidelines. Male, 4–8 week-old nude mice (Nu/J 002019, The Jackson Laboratory, Bar Harbor, Maine) were injected subcutaneously with MDA-MB 231 MFP or C2C12ras cells (1.5×10^6^). Tumors that were 30–100 mg in size formed 9 days to 4 weeks after grafting. Mice were fed low fluorescent chow 3 days prior to probe administration. Mice were injected either individually with a single activity based probe or ProSense probe or simultaneously with a mixture of ABP and ProSense probe labeled with different wavelength fluorophores. A constant amount of GB probes (25 nmols) and ProSense probes (2 nmol) was administered by tail vein injection (e.g. 1.2 mg/ml GB123, 2 mg/ml GB137, 1.6 mg/ml GB138 or 32 mg/ml ProSense 750 or ProSense 680). All probes were injected in solution of 67% DMSO 33% PBS in 100 µl total volume. Prior to imaging, mice were anesthetized with 3% isoflurane, starting prior to probe injection and up to 24 hr after injection. Tomographic images of mice were acquired using the FMT1 or FMT2500 imaging system using the 680 nm or 750 nm channels. Images are displayed as fluorescent tomography scans in color overlaid on an epifluorescent image in gray. The gain setting is kept constant for each probe throughout the scanning series. After the last imaging point, mice were humanely sacrificed and various organs were excised. Organs were then imaged *ex vivo* using the FMT2500. To quantify non-invasive images, total fluorescence (in pmol) was recorded in identically sized, 3 dimensional ROIs and normalized to tumor weight. Fluorescence measurements of Cy5 containing probes e.g. GB123, and GB137 were corrected to account for the use of a non-optimal filter of ex/em of 670/700 nm (the 680 channel) rather then the optimal 646/666 nm (Cy5 max Ex/Em). A correction factor was determined by measuring the ratio of the predicted fluorescence of a 100 pmol standard of GB123 with the 680 nm channel to the actual measurement of the standard (a predicted 100 pmols was measured as 16.3 pmol thus the scale factor was 6.12; Supplemental figure 1). Similarly, analysis of GB138 resulted in a scale factor of 2.84 for this fluorophore. The *ex vivo* organ fluorescence was measured using identical ROIs for each organ. Each ROI was smaller than the organs in size, and the maximum signal in each organ was recorded. Fluorescence of organs were normalized to the area of the ROI.

### Gel analysis of labeled tumors

Tumors from mice injected with probes were excised and flash frozen in liquid nitrogen. Tumors were lysed by dounce homogenization or by bead beating of tissue in cold buffer (1% Triton X-100, 0.1% SDS, 0.5% sodium deoxycholate in PBS). For radio-labeling studies 90 µg of protein in 30 µl of acetate buffer (50 mM sodium acetate pH 5.5, 5 mM MgCl_2_, and 4 mM DTT) was treated with 2 µl I^125^-JPM-OEt (4.6×10^6^ counts min^−1^ CPM) for 45 minutes. In addition, one control sample was pre-incubated for 15 minutes with GB111-NH_2_ (a cathepsin inhibitor)[4] prior to I^125^-JPM-OEt labeling. The reaction was stopped by addition of sample buffer (10% glycerol, 50 mM Tris HCl, pH 6.8, 3% SDS and 5% b-mercaptoethanol), boiled, and separated on a 12.5% SDS-PAGE gel. Wet gels were first scanned for fluorescence using an Odyssey scanner (LiCor Biosciences Nebraska USA) at 780/800 nm and then dried and exposed to a phosphor imager plate overnight. The plate was scanned with a Typhoon scanner.

## Supporting Information

Figure S1 (0.39 MB PDF)
