# Supplementary material for: Comparative Assessment of Substrates and Activity Based Probes as Tools for Non-Invasive Optical Imaging of Cysteine Protease Activity
Source: PLoS One. 2009 Jul 28;4(7):e6374. doi: 10.1371/journal.pone.0006374 (PMC2712068; doi:10.1371/journal.pone.0006374)
Supplement: Figure S1 — (0.39 MB PDF) [file pone.0006374.s001.pdf]

A

GB123<br>Measured pmol	Measured pmol	DMSO Control
17.76	16.84	1.04
14.24	13.32	0.80
19.74	18.82	0.92
	**16.33**	**avg**
	1.61	**std error**

100/average = correction factor

6.12

GB138<br>Measured pmol	Measured pmol	DMSO Control
32.81	31.89	1.04
39.45	38.53	0.80
36.12	35.20	**0.92**
	**35.21**	**avg**
	1.92	**std error**

100/average = correction factor

2.84

B

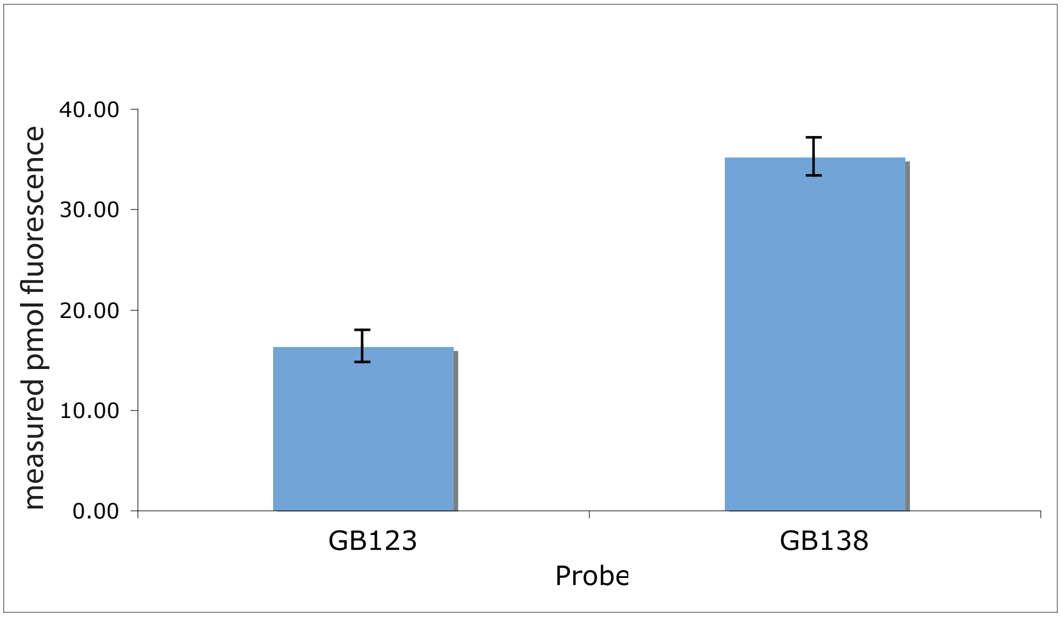

Figure S1. Determination of correction factors for Cy5 and IR800 probes. A. Values of pmol of fluorescence measured for 100 pmol of GB123 and GB138 probes loaded into a phantom and imaged with the FMT1 imaging system. Average and standard error are shown. Scale factors are shown in yellow. B. plot of average pmol of fluorescence for each probe with standard error shown.
